# Frailty Is Associated With Neutrophil Dysfunction Which Is Correctable With Phosphoinositol-3-Kinase Inhibitors

**DOI:** 10.1093/gerona/glaa216

**Published:** 2020-09-02

**Authors:** Daisy Wilson, William Drew, Alice Jasper, Helena Crisford, Peter Nightingale, Paul Newby, Thomas Jackson, Janet M Lord, Elizabeth Sapey

**Affiliations:** 1 MRC-Versus Arthritis Centre for Musculoskeletal Ageing Research, Institute of Inflammation and Ageing, University of Birmingham, Queen Elizabeth Hospital Birmingham, UK; 2 Birmingham Acute Care Research Group, Institute of Inflammation and Ageing, University of Birmingham, Queen Elizabeth Hospital Birmingham, UK; 3 NIHR Clinical Research Facility, University Hospitals Birmingham NHS Foundation Trust, UK; 4 NIHR Birmingham Biomedical Research Centre, University Hospitals Birmingham NHS Foundation Trust and University of Birmingham, UK

**Keywords:** Comorbidity, Inflammation, Innate immunity, Proteinases

## Abstract

Neutrophil dysfunction has been described with age, appears exaggerated in infection, with altered phosphoinositol signaling a potential mechanism. However, functional aging is heterogeneous. Frailty is a negative health status and is more common in older adults. We hypothesized that neutrophil migration may be compromised in frailty, associated with the degree of frailty experienced by the older person. We compared measures of frailty, neutrophil function, and systemic inflammation in 40 young and 77 older community-dwelling adults in the United Kingdom. Systemic neutrophils exhibited an age-associated reduction in the accuracy of migration (chemotaxis) which was further blunted with frailty. The degree of migratory inaccuracy correlated with physical (adjusted hand grip strength) and cognitive (Stroop test) markers of frailty. Regression analysis demonstrated that age, Charlson comorbidity index, and frailty index were able to predict neutrophil chemotaxis. Reduced chemotaxis of neutrophils from frail adults could be reversed using selective PI3K inhibitors. Exposure of neutrophils from young adults to plasma from chronically inflamed frail older adults could not recapitulate the migratory deficit in vitro, and there were no relationships with systemic inflammation and neutrophil dysfunction. Frailty exaggerated the neutrophil deficits seen with advanced age but aspects of the frailty-associated deficit in neutrophil function are rescuable and thus potentially form a therapeutic target to improve outcomes from infection in older adults.

There is increasing interest in therapeutic strategies to maintain health with advancing age. Frailty is a manifestation of unhealthy aging (1) and an independent predictor of poor outcomes (2). Frailty is distinct from physiological aging, disability, or comorbidity, although more common in older people with disability and comorbidity (3). Clinically, frailty is identified using composite scores, including Rockwood’s Frailty Index (FI) (4).

Inflammation and immune cell function have been implicated in the pathophysiology of frailty (5). Frailty appears associated with an increase in systemic inflammation (6), although this has not been consistently replicated (7). Adaptive immune cell subtypes have been shown to be altered in frailty (8), with less known about innate immune cells such as neutrophils.

The susceptibility to infections increases with both age and frailty (9,10). Neutrophils are of primary importance during infective challenges, and previous work by our group and others have described neutrophil dysfunction with an individual’s increasing age. This includes reduced phagocytosis to some but not all pathogens (11), a failure to prevent apoptosis in the presence of inflammatory stimuli, reduced neutrophil extracellular trap formation (12), and inaccurate migration (13,14). These altered functions have been postulated to represent a marker of biological age (15).

Two interwoven factors may contribute to the mechanism of reduced neutrophil migratory accuracy. Increased intracellular Class 1 delta(δ) and gamma(γ) phosphoinositol-3-kinase (PI3K) signaling has been implicated in inaccurate neutrophil migration with age, which in vitro selective PI3K inhibitors can restore (16). The systemic inflammation associated with aging and frailty might alter neutrophil behaviors, making the cells less responsive to chemotactic cues seen during inflammation and infection.

We hypothesized that frailty would be associated with inaccurate neutrophil migration and an increased inflammatory burden. We further hypothesized that PI3K signaling would be implicated in altered neutrophil migration.

This study had 2 aims. First, to determine whether neutrophil migration was altered in cells isolated from frail older adults compared with healthy older and younger adults. Second, to assess the mechanism of effect including whether PI3K signaling was implicated in migratory function and thus could form a therapeutic target and/or whether an inflammatory systemic environment could induce compromised migratory behavior.

## Materials and Methods

### Study Participants

One hundred and seventeen participants were recruited to this study and divided into 3 groups: healthy younger adults (HY), healthy older adults (HO), and frail older adults (FO). See Supplementary Material for details. All participants were recruited between 2015 and 2019.

Healthy younger adults were aged older than 18 and younger than 35 years and HO were aged older than 65 years. Frail older adults were aged older than 65 years, had a FI greater than 0.2 (4). Supplementary Table S1 provides a full list of exclusion and inclusion criteria. All participants were clinically characterized by a single assessor who was clinically qualified and an expert in geriatric medicine. These assessments were used to form the FI, as described in Supplementary Table S2.

### Isolation of Blood Neutrophils and Neutrophil Migration

For full methods see Supplementary Methods. In brief, neutrophils were isolated from whole blood and were more than 95% pure, more than 97% viable, by the exclusion of trypan blue, and migration was assessed using an Insall chamber (Weber Scientific International Ltd, UK), as described previously (17). About 10 nM *N*-formylmethionine-leucyl-phenylalanine (fMLP) and 100 nM interleukin 8 (CXCL8) were used as chemoattractants or appropriate vehicle control. Phosphoinositol-3-kinase isoform-selective inhibitors (IC_50_ concentrations) included: Class 1δ (Cal-101 75 nM; Selleck, Cambridge, UK), γ (AS-252424 33 nM; Selleck), or vehicle control and were incubated with neutrophils for 45 minutes prior to migration. Neutrophils from HY were studied following 45-minute incubation with pooled plasma (PP) from 18 HO (mean FI: 0.04) or 18 FO (mean FI: 0.41) following washing and resuspending in Roswell Park Memorial Institute-1640 medium (Sigma–Aldrich, UK). A 250 µL aliquot of plasma was combined from each individual sample to form the pool which was used for all experiments. Donors of PP samples were representative of the cohorts as a whole with no significant differences in demographics. All analysis was carried out by a single analyst utilizing a randomization method for initial cell selection. Images were captured by a Leica DMI6000B with DFC360FX camera as described previously (18). The images were analyzed using ImageJ software (Wayne Rasband, NIH, USA).

Two migratory parameters were assessed: mean cell speed of movement, defined as the distance traveled between frames in any direction over time (termed chemokinesis) and mean cell velocity, defined as the speed in a consistent direction toward the chemoattractant (termed chemotaxis) (17), both measured in micrometers per minute.

### Neutrophil Elastase Activity

Aα-Val360, a neutrophil elastase (NE)-specific fibrinogen degradation product and a surrogate marker of NE activity in vivo, was measured in plasma as described previously (19).

### Cytokine and High Sensitivity CRP Plasma Concentration

Inflammatory mediators were measured in plasma using commercially available kits, as per manufacturer’s instructions (Bio-Plex Pro Human Cytokine Standard 27-Plex and Bio-Rad; hsCRP kit, IBL International).

### Statistics

Statistical analyses were performed using SPSS (version 22.0; IBM Corp., USA). Data were tested for a normal distribution using the Shapiro–Wilk or Kolmogorov–Smirnov test and the appropriate parametric (analysis of variance, post hoc Tukey’s, Pearson correlation [shown as *r*], Kruskal–Wallis with Dunn’s multiple comparison test, Wilcoxon signed-rank test, or Spearman’s rank correlation [shown as “rho”]). Linear regression modeled if chemotaxis predicted FI. A *p* value of less than .05 was considered to be statistically significant. All *p* values are reported and all tests are described in the text or table/figure legends with adjustment for multiple comparisons where stated.

### Data Sharing Statement

For original data, please contact d.v.wilson@bham.ac.uk.

## Results

All participants gave their informed written consent following approval from the Health Research Authority and Research Ethics Committee (15/WM/0002). Table 1 describes participant demographics. For all results, frail older adults are referred to as FO, healthy old adults as HO, and healthy young as HY.

**Table 1.** Demographics of Healthy Young Adults, Healthy Older Adults, and Frail Older Adults

		HY	HO	FO	Group Comparison	Pairwise Comparison
Number		40	40	37		
Gender % (F:M)		50:50	67:33	54:46	*p* = .330	
Age		26.0 (21.3–31.0)	71.0 (70.0–79.0)	84.0 (73.8–88.5)	***p* < .001**	**HY-HO *p* < .001**
						**HY-FO *p* < .001**
						**HO-FO *p* = .019**
Comorbid conditions (%)	0	100	77.5	34.3	***p* < .001**	HY-HO *p* = .124
	1		20	40		
	2		2.5	14.3		**HY-FO *p* < .001**
	3			11.4		**HO-FO *p* < .001**
Frailty index		0.02 (0.00–0.02)	0.04 (0.02–0.08)	0.30 (0.25–0.36)	***p* < .001**	**HY-HO *p* = .005**
						**HY-FO *p* < .001**
						**HO-FO *p* < .001**
Adjusted grip strength		1.71 (1.61–1.81)	1.31 (1.23–1.39)	0.73 (0.64–0.82)	***p* < .001**	**HY-HO *p* < .001**
						**HY-FO *p* < .001**
						**HO-FO *p* < .001**
Walk speed (m/s)		1.62 (1.34–1.79)	1.26 (1.17–1.43)	0.33 (0.19–0.50)	***p* < .001**	**HY-HO *p* = .007**
						**HY-FO *p* < .001**
						**HO-FO *p* < .001**
SPPB		12.0 (12.0–12.0)	12.0 (10.0–13.0)	2.0 (1.0–4.0)	***p* < .001**	HY-HO *p* = .051
						**HY-FO *p* < .001**
						**HO-FO *p* < .001**

*Notes:* The demographics of the 3 different groups are described. HY = healthy young adults (*n* = 40), HO = healthy older adults (*n* = 40), and FO = frail older adults (*n* = 37). Categorical data are presented as proportions of the total. Age, frailty index, walk speed, and short physical performance battery (SPPB) are presented as the median and interquartile range in parentheses. Adjusted grip strength is presented as mean with 95% confidence intervals and compared between groups using an ANOVA with post hoc Tukey’s for pairwise comparison. Gender was assessed using a chi-squared test and all other statistical tests are Independent Kruskal–Wallis with Dunn’s pairwise comparison adjusted with Bonferroni correction. All statistically significant values appear in bold text.

### Neutrophil Chemokinesis Is Preserved With Frailty But Chemotaxis Is Reduced

There were no significant differences in the speed of migration in any direction (chemokinesis) of isolated neutrophils to fMLP or CXCL8 between the 3 groups, FO, HO, or HY (Supplementary Figure S1A and C). There was a reduction in chemotaxis toward fMLP across the groups (Independent Kruskal–Wallis *p* < .0001) where chemotaxis was reduced in HO (Dunn’s multiple comparison test, *p* < .0002) and FO (Dunn’s multiple comparison test, *p* < .0001) compared with HY. Neutrophils from HO and FO were less accurate in their migratory pathways toward fMLP and CXCL8 than those isolated from young adults. Frail older adults neutrophils showed a reduction in chemotaxis to CXCL8 compared to HO but not fMLP (Supplementary Figure S1B and D).

### Neutrophil Chemotaxis Relates to Physical and Cognitive Parameters of Frailty

**Figure 1.**
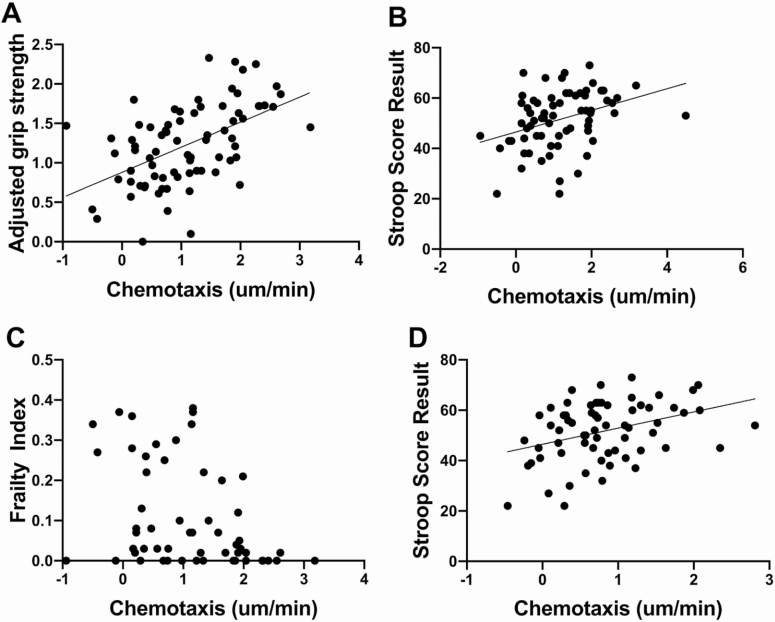
Migration of peripherally isolated neutrophils and relationship with frailty parameters. Neutrophils isolated from healthy young adults (HY), healthy older adults (HO), and frail older adults (FO) were migrated toward *N*-formylmethionine-leucyl-phenylalanine (fMLP) or CXCL8. Each dot represents neutrophil migration for one person. There were relationships between migration toward fMLP and (**A**) adjusted hand grip, *r* = 0.5239, *p* < .0001; (**B**) Stroop score result, *r* = 0.359, *p* = .0025; (**C**) Frailty index rho = −0.401, *p* = .002. There were also relationships between migration toward CXCL8 and (**D**) Stroop score result, *r* = 0.364, *p* = .0026. Pearson’s correlation coefficient for A, B, and D. Spearman’s correlation for C.

When HY, HO, FO participants were included together, chemotaxis correlated positively with grip strength (adjusted for gender and BMI (18)) and Stroop score and negatively with overall FI. For migration to fMLP: adjusted hand grip, *r* = 0.5239, *p* < .0001 (Figure 1A); Stroop score, *r* = 0.359, *p* = .0025 (Figure 1B); and FI, rho = −0.4012, *p* = .002 (Figure 1C). For migration to CXCL8: adjusted hand grip, *r* = 0.215, *p* = .08; Stroop score, *r* = 0.364, *p* = .0026 (Figure 1D); and FI, rho = −0.312, *p* = .04. There were no significant relationships between other frailty parameters including the short physical performance battery and Addenbrooke’s cognitive examination and neutrophil chemotaxis.

### Neutrophil Chemotaxis Relates to the Charlson Comorbidity Index and Predicts Frailty

When HY, HO, FO participants were included together, there was a negative relationship between Charlson comorbidity index (CCI) and chemotaxis toward fMLP (Spearman’s rho −0.423, *p* < .0001) and CXCL8 (rho −0.414, *p* = .0003) but not chemokinesis (fMLP: Spearman’s rho = −0.1992, *p* = .08; CXCL8 rho −0.21, *p* = .06).

Linear regression modeling demonstrated that chemotaxis toward CXCL8 and fMLP could be predicted by age, frailty, and CCI (standardized β: age = −0.301, *p* = .013; FI = −0.294, *p* = .015; CCI = −0.253, *p* = .037).

### Selective PI3K Inhibitors Improve Migratory Accuracy in Neutrophils From FO Adults

**Figure 2.**
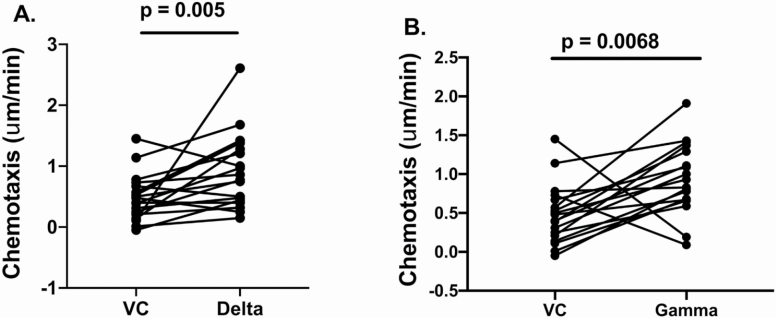
PI3K inhibition restores neutrophil migratory accuracy in frail older adults. Neutrophils isolated from frail older adults were migrated toward CXCL8 100 nM following incubation with vehicle control (VC; Roswell Park Memorial Institute-1640 medium and Dimethyl sulfoxide) or the PI3Kδ (**A**) or PI3Kγ inhibitors (**B**). Each dot represents neutrophil migration for one person. Chemotaxis (migratory accuracy) is shown and expressed as µm/min. The frailty-associated reduction in chemotaxis was improved following incubation with PI3K inhibitors δ and γ (*p* values as given, Wilcoxon signed-rank test for both).

FO neutrophil chemotaxis toward CXCL8 increased following incubation with Class 1δ and γ PI3K inhibitors. Median and interquartile range (IQR) for all: vehicle control: 0.44 μm/min (0.19–0.68) versus δ 0.81 μm/min (0.43–1.31), Wilcoxon test, *p* = .005, and γ 0.88 μm/min (0.67–1.31), Wilcoxon test, *p* = .0068 (Figure 2).

The PI3K inhibitor-associated improvement in chemotaxis related to both FI (with individuals with greater frailty showing greater improvements from baseline) and adjusted hand grip strength (with individuals with low grip strength [adjusted for gender and BMI] having greater improvements from baseline) when expressed as a fold rather than an absolute change. Spearman’s correlation between the fold change in chemotaxis, vehicle control to PI3Kδ and FI, rho =0.452. *p* = .001; fold change in chemotaxis, vehicle control to PI3Kδ and adjusted hand grip, rho = −0.509, *p* < .001 and fold change in chemotaxis, vehicle control to PI3Kγ and FI, rho = 0.435, *p* = .001; fold change in chemotaxis, vehicle control to PI3Kγ and adjusted hand grip, rho = −0.388, *p* = .004.

### Systemic NE Activity Footprint Is Reduced in Frailty

Neutrophil migration through tissue is achieved through the release of the protease NE. AαVal360 measures the activity of NE in vivo, a biomarker of chemotactic accuracy and neutrophil activity. Systemic NE activity differed across groups. AαVal360, median (IQR) for all: HY 8.75 nM (6.35–9.93) versus HO 16.50 nM (14.00–19.00) versus FO 9.50 nM (7.00–13.00), Kruskal–Wallis *p* < .0001. AαVal360 was higher in HO compared to FO (*p* = .0003) and HO compared to HY (*p* < .0001), but there were no differences between HY and FO (*p* = .783), Dunn’s multiple comparison test.

### Systemic Inflammation in Frailty

The concentration of high sensitivity CRP was increased in FO adults compared to both HY and HO adults (Supplementary Figure S2). There was no correlation between neutrophil chemotaxis and high sensitivity CRP concentration (Pearson’s correlation *r* = −0.239, *p* = .07).

For cytokine measurement, only results where more than 50% of values were above the minimum level of detection are reported (16 of 27 inflammatory mediators). In these mediators, 7 had comparable concentrations between the 3 groups, 7 were higher in both older adult groups compared with young adults (IL-ra, IL-4, CXCL8, IL-17, Eotaxin, IP10, MIP1a). MCP1 and IL-17 were lower in FO than HO groups and MIP1a was higher in FO adults compared to HO adults (Supplementary Table S3).

### The Frail Neutrophil Functional Phenotype Is Not Inducible by Plasma From Frail Adults

Neutrophils from 11 HY adults were incubated with PP made up from 18 FO (FO-PP), 18 HO (HO-PP), or 18 HY (HY-PP), and migration experiments were repeated.

Incubation with FO-PP or HO-PP did not change the HY neutrophils migratory speed or accuracy toward CXCL8 compared to HY neutrophils being incubated with HY-PP. Chemokinesis, median (IQR): HY-PP 2.51 μm/min (2.18–3.49) versus HO-PP 3.63 μm/min (2.83–4.17) versus FO-PP 3.29 μm/min (2.43–4.93), Friedman test *p* = .420. Chemotaxis, median (IQR): HY-PP 1.68 μm/min (0.45–2.81) versus HO-PP 1.13 μm/min (0.96–2.01) versus FO-PP 0.66 μm/min (0.31–2.87), Friedman test, *p* = .317.

## Discussion

This study presents novel data on the differential effects of age and frailty on neutrophil function, linking migratory accuracy with global markers of function (grip strength and cognitive ability). Frailty was also associated with reduced migratory accuracy toward CXCL8 compared with healthy young and old adults. This phenotype could not be induced by exposing neutrophils from young adults to frail older plasma, perhaps supporting an intrinsic deficit, but was rescuable using selective PI3Kγ and PI3Kδ inhibitors.

This study is of importance. It highlights potential cellular mechanisms for reduced neutrophil migration which are targetable therapeutically. Inhibition of PI3Kδ and PI3Kγ restored the chemotactic ability of neutrophils from frail older adults, offering the possibility of modifying the innate immune system during infections in frail older adults to improve responses. The PI3K pathway could be targeted at differing entry points. For example, simvastatin inhibits GTPases (20) which represent a downstream effector of PI3K signaling, and adjuvant statins were associated with both improvements in neutrophil migratory accuracy and survival benefit in hospitalized patients with community-acquired pneumonia in a cohort of older, frail adults (14). The negative relationship between grip strength and fold change improvement in chemotaxis following PI3K inhibition further highlights the close relationship between frailty and immune aging (immunosenescence) and may help clinically identify a suitable population for this therapeutic intervention.

The reduction in AαVal360 in frail older adults compared to healthy older adults is of interest. Previous studies from our group report AαVal360 levels of approximately 7.5 nM in young adults and approximately 17 nM in healthy older adults (16), supporting increased neutrophil activity with age, mirroring the levels described in the current study. The reduced systemic neutrophil proteinase activity in frailty alongside reduced migratory accuracy might suggest a double insult to cell function, impeding bacterial killing further. Although the previous investigation of frailty-associated immunosenescence is limited, systemic neutrophils isolated from frail older adults were shown to produce fewer chemokines than neutrophils from healthy older adults (21). This requires further study at baseline and following inflammatory challenges in vitro and in vivo to confirm this interpretation.

A second key finding is the heterogeneity of systemic inflammation in frailty. In a systematic review of inflammation in more than 3000 frail adults, the variance in inflammatory mediators was noted, including differences between the findings of longitudinal and cross-sectional studies (7). It is likely that there will be different cohorts of frail adults, some with or without comorbidities, with only a proportion demonstrating a pro-inflammatory phenotype. However, as the FO neutrophil migration phenotype was not inducible by acute exposure to plasma from FO adults, it is likely that the change in neutrophil function is not merely a response to systemic inflammation alone. This suggests that anti-inflammatory strategies may not be sufficient to improve cell function.

Third, it highlights the need for careful characterization of patients when studying the effects of age or immunosenescence. Here frailty, age, and comorbid disease burden all related to the accuracy of neutrophil migration. Another study investigating neutrophil chemotaxis in healthy older adults grouped by level of daily physical activity reported similar heterogeneous results, with activity positively correlating with migratory accuracy (22). Previous studies (16,23,24) have demonstrated a reduction in migratory accuracy with age but only considered the impact of specific diseases and did not consider the potential effect of frailty or comorbidity.

There are a number of important limitations to this work. The frail older adults were older than the healthy older adults. These data are cross-sectional and therefore cannot comment on the time course between the onset of frailty and the reduced migratory phenotype. As physical activity appears to affect neutrophil migratory accuracy (22), it is possible that reduced physical activity in the frail older adults was a contributor, but we did not assess daily physical activity objectively in our cohorts, instead using self-reported levels of activity as a part of the frailty assessment. We did not look specifically at each component of the FI to see what characteristics might most predict neutrophil dysfunction. Isolated neutrophils were used for these studies. The process of isolation can affect on the cell; however, the current study finds clear differences between frail old, healthy old, and young adults’ neutrophils under the same conditions. The Insall chamber, while allowing single-cell migration to be assessed, does not provide the same migratory platform as is found in vivo and more physiological models should now be assessed. Not all aspects of migration were assessed, including responses to different chemoattractant concentrations over different time courses. Not all neutrophil functions were studied due to experimental constraints using these short-lived cells. Although mechanisms were suggested by association, these are not confirmed and in-depth studies are required. Although PI3K inhibitors have been associated with improved neutrophil migration in vitro, the effects of PI3K inhibition on other cellular functions should be assessed. This study utilized systemic neutrophils, and neutrophil functions may alter during transmigration. Studying transmigrated cells during particular infections or inflammatory events would be of interest.

In summary, neutrophil chemotaxis is compromised with age and is further challenged with frailty. Neutrophils from frail adults appear to represent an exaggerated version of neutrophils from healthy older adults, but with some distinct characteristics (such as reduced evidence of degranulation) which are not inducible by inflammatory cues but are correctable by PI3K inhibition. Frail older adults are known to suffer from more infections and experience worse outcomes from these infections. Targeting neutrophil functions may provide a new therapeutic strategy for this vulnerable group.

## Supplementary Material

glaa216_suppl_Supplementary_Material
