# Supplementary material for: Frailty Is Associated With Neutrophil Dysfunction Which Is Correctable With Phosphoinositol-3-Kinase Inhibitors
Source: J Gerontol A Biol Sci Med Sci. 2020 Sep 2;75(12):2320–5. doi: 10.1093/gerona/glaa216 (PMC7662170; doi:10.1093/gerona/glaa216)
Supplement: glaa216_suppl_Supplementary_Material [file glaa216_suppl_supplementary_material.docx]

**Frailty is associated neutrophil dysfunction which is correctable with phosphoinositol-3-kinase inhibitors**

**Daisy Wilson, William Drew, Peter Nightingale, Paul Newby, Thomas Jackson, Janet M Lord, Elizabeth Sapey**

**ONLINE SUPPLEMENT**

**Supplementary Methods**

**Subject participants**

HY adults were recruited from staff and students from the University of Birmingham, HO were recruited from the 1000 Elders cohort. FO were recruited from the 1000 Elders cohort, patients attending Geriatric outpatient clinics and medically stable patients with no evidence of ongoing infection in the University Hospitals Birmingham NHS Foundation Trust awaiting social care placements. The 1000 Elders Cohort is a research cohort of older adults in the West Midlands who have consented to be contacted about ethically approved studies into ageing.

**Patient Characterisation**

**Stroop test**

Stroop is a commonly used psychological test that tests the ability to process information and respond appropriately (1). In brief, the participant is asked to read a list of words as quickly as possible without making any mistakes; the words are either the word red, blue or green. The participant is then asked to name the colour of lines of crosses which are printed in either red, blue or green. Finally the participant is asked to name the colour of ink a word is printed in ignoring the written word. For example, the participant should say green for the word blue printed in green. The raw data from these three individual assessments are combined to reach the final score.

**SPPB assessment**

The short physical performance battery (SPPB) is a group of measures that combines the results of the gait speed, chair stand and balance tests (2). It has been used as a predictive tool for possible disability and can aid in the monitoring of function in older people. The scores range from 0 (worst performance) to 12 (best performance). The SPPB has been shown to have predictive validity showing a gradient of risk for mortality, nursing home admission, and disability.

**Neutrophil migration**

Neutrophil samples were suspended at 2x10^6^ cells/ml in Roswell Park Memorial Institute (RPMI) 1640 media (Thermofisher, UK) with 0.15% (v/v) of 7.5% Bovine serum albumin (BSA) (Sigma-Aldrich, Dorset, UK). Sterilised coverslips were coated in 400μl 7.5% BSA. The excess was discarded and 400μl of incubated neutrophils added. The Insall chamber chemoattractant wells were filled with 70μl of chemoattractant or negative control. The field of vision was filmed for 20 minutes, with an image captured every 20 seconds.

Each image sequence was exported from Leica as AVI files and imported to ImageJ. The manual tracking plugin was used to track the movement of the neutrophils in the visual field, Neutrophil were chosen at random for assessment using a grid and random grid reference generator(3, 4).

**Supplementary Tables**

**Table S1. Inclusion and exclusion criteria for recruitment to study**

| **Group** | **Age** | **Clinical Criteria** |
| --- | --- | --- |
| Healthy Young Adults  Healthy Older Adults | 18-35  >65 | 1. Able to provide informed written consent. 2. Absence of  - Significant respiratory symptoms including breathlessness and cough - Diagnosis of chronic inflammatory disease including Diabetes, COPD, Asthma, Interstitial Lung disease, Bronchiectasis - Haematological or solid organ malignancy - Immunosuppressive medications |
| Frail Older Adults | >65 | 1. Able to provide informed written consent 2. Identified as frail on Frailty Index   (FI> 0.2)   1. Absence of  - Haematological or solid organ malignancy - Immunosuppressive medications |

**Legend.**  The inclusion and exclusion criteria for the recruitment of the three groups, healthy young adults, healthy older adults and frail older adults, to the study.

Table S2. Frailty Index Components

| **Domain** | **FI Variable** | **Scoring Criteria** |
| --- | --- | --- |
| Medical | Stroke | Y=1, N=0 |
|  | Myocardial Infarction | Y=1, N=0 |
|  | Congestive Cardiac Failure | Y=1, N=0 |
|  | Diabetes Mellitus | Y=1, N=0 |
|  | COPD | Y=1, N=0 |
|  | Cancer | Y=1, N=0 |
|  | Number of medications | >10=1, 4-10=0.5, <4=0 |
|  | Continence | Partially/totally incontinent=1, Lose control of urine when don’t want to =0.5, No concerns with continence = 0 |
|  | Self-reported health | 1,2 = 1, 3=0.5, 4,5= 0 (Scale of 1-5. 1 being poor and 5 being excellent) |
|  | Self-reported weight loss – in the last year have you lost more than 4.5kg? | Y=1, Don’t know but clothing is looser = 0.5, N=0 |
|  | BMI | <18.5 or >30=1, 25-30=0.5 |
|  | Physician assessment of Clinical Frailty Scale | 7,8=1, 6=0.75, 5=0.5, 4=0.25, 1,2,3=0 |
|  | Food intake | Severe decrease=1, Moderate decrease=0.5, No decrease=0 |
| Psychological | Self-reported low mood - do you often feel sad or depressed? | Y=1, N=0 |
| Independence | Bathing | Needs help =1, Independent=0 (use Katz scoring system but reversed) |
|  | Dressing | Needs help =1, Independent=0 (use Katz scoring system but reversed) |
|  | Transferring | Needs help =1, Independent=0 (use Katz scoring system but reversed) |
|  | Feeding | Needs help =1, Independent=0 (use Katz scoring system but reversed) |
|  | Toileting | Needs help =1, Independent=0 (use Katz scoring system but reversed) |
|  | Finances | Needs help =1, Independent=0 (use Lawton scoring system but reversed) |
|  | Shopping | Needs help =1, Independent=0 (use Lawton scoring system but reversed) |
|  | Housework | Needs help =1, Independent=0 (use Lawton scoring system but reversed) |
|  | Meal Preparation | Needs help =1, Independent=0 (use Lawton scoring system but reversed) |
|  | Medications | Needs help =1, Independent=0 (use Lawton scoring system but reversed) |
| Cognition | Addenbrooke’s Cognitive Examination | <82=1, >82=0 |
| Physical Function | Walk Speed | Frailty phenotype ‘slowness’ positive (adjusted for height and gender) =1, <0.8=0.5, >0.8=0 |
|  | Falls in last year | Y=1, N=0 |
|  | Everything is an effort | Most of the time =1, Moderate amount of time =0.75, Some of the time =0.5, None of the time =0 |
|  | Trouble getting going | Most of the time =1, Moderate amount of time =0.75, Some of the time =0.5, None of the time =0 |
|  | Handgrip | Lowest 20% for gender/BMI = 1, Highest 80% for gender/BMI =0 |

**Legend.** The variables included in the Frailty Index and the criteria for scoring. Values for all variables summed together and divided by 30 to give a compound continuous measure of frailty. Frailty defined as a score greater than 0.2.

**Table S3. Systemic Inflammatory mediators in each group**

| **Mediator** | **HY**  **(n = 28)** | **HO**  **(n = 38)** | **FO**  **(n = 34)** | **Overall difference** | **Pairwise comparisons** |
| --- | --- | --- | --- | --- | --- |
| IL-1ra  (pg/ml) | 49.58  (33.10 – 75.39) | 64.71  (55.36 - 124.30) | 78.95  (64.71 – 95.81) | p < 0.0001 | HY: HO p = 0.0033  HO: FO p > 0.999  HY: FO p = 0.0017 |
| IL-4  (pg/ml) | 5.71  (4.67 – 7.99) | 10.42  (8.61 – 13.15) | 9.17  (4.60 – 13.25) | p = 0.001 | HY: HO p =0.0006  HO: FO p = 0.370  HY: FO p = 0.083 |
| IL-7  (pg/ml) | 12.04  (8.38 – 16.02) | 16.02  (12.04 – 18.30) | 14.05  (7.84 – 17.78) | p = 0.114 |  |
| CXCL8  (pg/ml) | 0.01  (0.01 – 5.51) | 7.37  (5.28 – 8.94) | 9.22  (4.57 – 14.79) | p < 0.0001 | HY: HO p <0.0004  HO: FO p > 0.999  HY: FO p < 0.0001 |
| IL-9  (pg/ml) | 52.87  (46.78 – 57.27) | 47.02  (23.99 – 62.48) | 47.51  (0.1 – 53.85) | p = 0.213 |  |
| IL-17  (pg/ml) | 0.01  (0.001 – 16.06) | 19.75  (13.43 – 26.07) | 16.06  (0.1 – 21.65) | p = 0.0011 | HY: HO p = 0.0002  HO: FO p = 0.0063  HY: FO p = 0.04 |
| Eotaxin (pg/ml) | 102.80  (72.01 – 140.91) | 167.30  (167.30 – 213.31) | 152.2  (96.25 – 203.1) | p = 0.0011 | HY: HO p = 0.0008  HO: FO p = 0.8133  HY: FO p = 0.034 |
| FGF  (pg/ml) | 17.16  (17.16 – 20.09) | 20.49  (17.16 – 23.44) | 17.16  (0.1 – 23.44) | p = 0.085 |  |
| IP10  (pg/ml) | 877.70  (677.81 – 1270.00) | 1440  (1064 – 2069) | 1467  (972.7 – 1860) | p = 0.0002 | HY: HO p = 0.0002  HO: FO p > 0.999  HY: FO p = 0.0052 |
| MCP1  (pg/ml) | 25.75  (18.60 – 31.46) | 30.69  (20.93 – 44.93) | 19.77  (12.38 - 35.35) | p < 0.0001 | HY: HO p = 0.094  HO: FO p = 0.018  HY: FO p = 0.358 |
| MIP1a  (pg/ml) | 0.97  (0.79 – 1.41) | 1.41  (1.11 – 2.46) | 2.79  (1.94 – 3.75) | p< 0.0001 | HY: HO p = 0.0425  HO: FO p = 0.0053  HY: FO p <0.0001 |
| MIP1b  (pg/ml) | 2120  (1742 – 2480) | 1760  (1198 – 5693) | 1807  (964.6 – 3196) | p = 0.350 |  |
| PDGF  (pg/ml) | 82.46  (77.98 – 92.99) | 82.26  (66.25 – 140.20) | 84.61  (69.54 – 99.04) | p = 0.982 |  |
| RANTES (pg/ml) | 12491  (10742 – 12518) | 12443  (8109 – 21790) | 11320  (7434 – 13939) | p = 0.201 |  |
| TNFα  (pg/ml) | 36.90  (32.78 – 41.00) | 41.00  (32.78 – 121.37) | 38.95  (31.74 – 74.52) | p = 0.301 |  |
| CRP  (μg/ml) | 1.39  (0.52 – 2.38) | 1.35  (0.58 – 2.23) | 8.87  (2.03 – 10.00) | p < 0.0001 | HY: HO p = 0.925  HO: FO p < 0.0001  HY: FO p < 0.0001 |

**Legend**. Abbreviations: IL= Interleukin; IL-1ra = Interleukin 1 Receptor Antagonist; FGF = Fibroblast growth Factor; IP10 = Interferon gamma induced protein 10; MCP-1 = Monocyte Chemoattractant Protein-1; MIP = Macrophage Inflammatory Protein; PDGF = Platelet Derived Growth Factor; RANTES = Regulated upon Activation, Normal T cell Expressed, and Secreted; TNF = Tumour Necrosis Factor; CRP = C Reactive Protein. Results were only included for analysis where sample values were within the reportable range of the assay. Numbers included in each group are given. All results are presented as median and IQR. Overall difference has been assessed using Kruskal-Wallis with post-hoc corrected Dunn’s pairwise comparison

**Supplementary Figures**


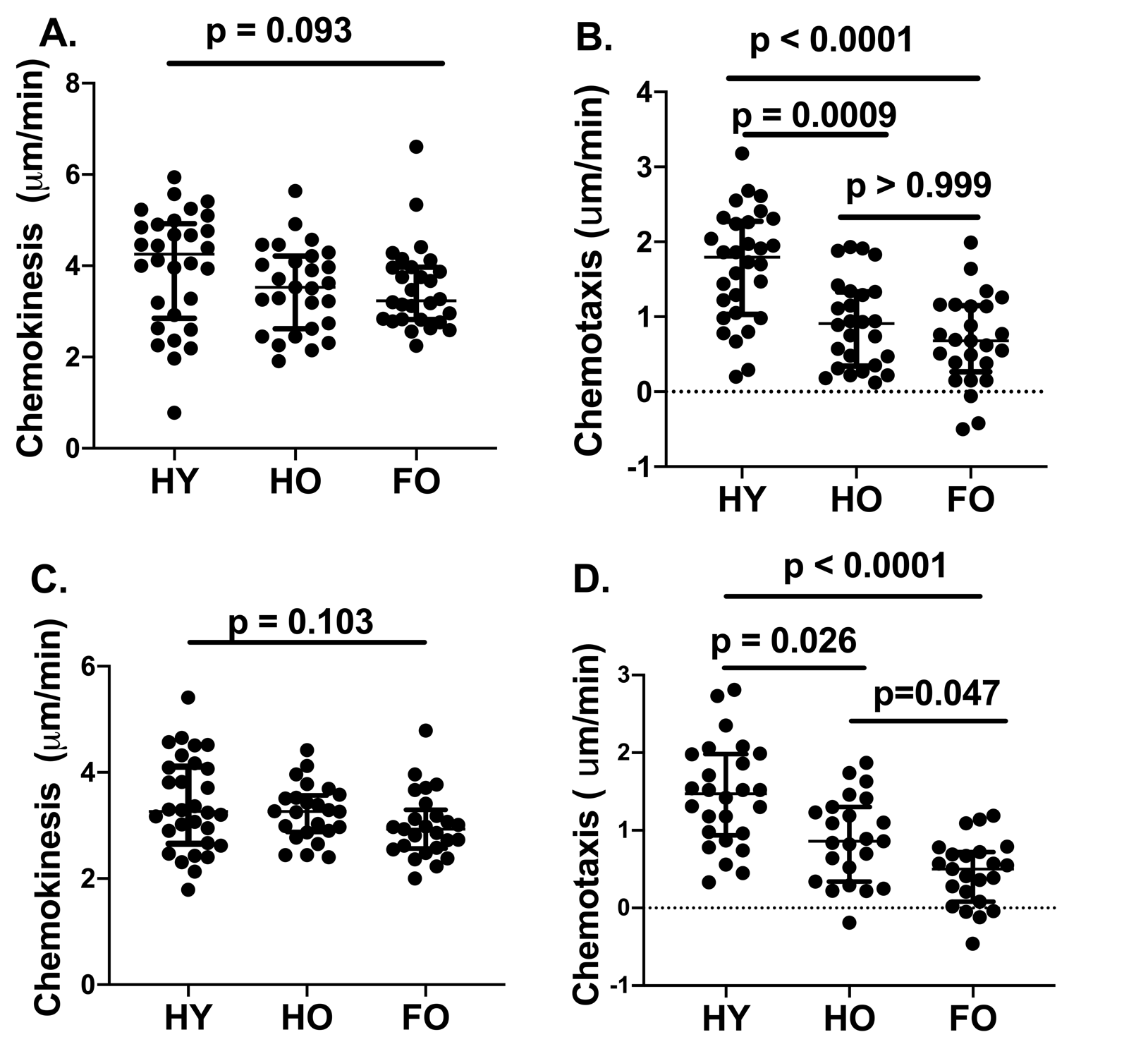


**Figure S1. Migration of peripherally isolated neutrophils and relationship with frailty parameters**

Neutrophils isolated from healthy young adults (HY), healthy older adults (HO) and frail older adults (FO) were migrated towards fMLP or CXCL8. Each dot represents neutrophil migration for one person. Chemokinesis is shown in figure **1A** and **1C** and chemotaxis in figure **1B** and **1D,** all expressed as µm/min. The line and error bars are median and interquartile range for all figures.

There were no differences in chemokinesis between groups (Independent Kruskal-Wallis: 1A: p=0.093, 1C: p = 0.103). 1B: There was a reduction in chemotaxis towards fMLP across the groups (Independent Kruskal-Wallis p < 0.0001). Chemotaxis was reduced in HO (Dunn’s multiple comparison test, p < 0.0002) and FO (Dunn’s multiple comparison test, p < 0.0001) compared with HY. 1D: there was a reduction in chemotaxis towards CXCL8 across the groups (Independent Kruskal-Wallis p < 0.0001). Chemotaxis was reduced in HO (Dunn’s multiple comparison test, p = 0.026) and FO (Dunn’s multiple comparison test, p = 0.047) compared with HY.


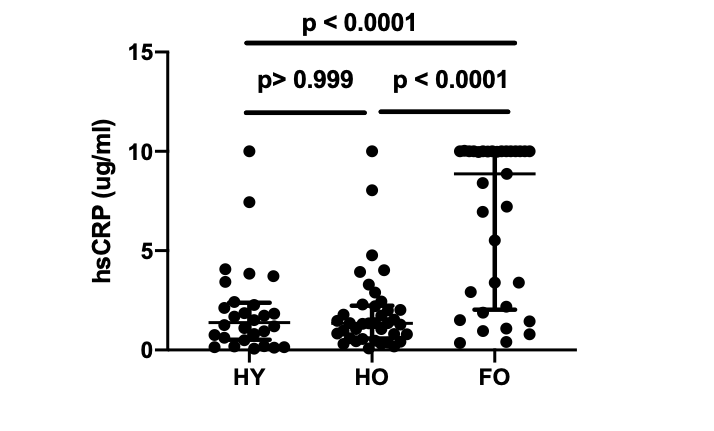


**Figure S2. Concentration of the acute phase protein hsCRP in serum.**

**Legend.** Concentration of hsCRP (µg/ml) was measured using a commercially available ELISA in healthy younger adults (HY, n=28), healthy older adults (HO, n=38) and frail older adults (FO, n=33). Each dot is one person. The line and error bars represent median (IQR). There were differences between groups (Kruskal Wallis test p < 0.0001). Dunn’s multiple comparison tests showed no difference between HY and HO (p > 0.999) but significant differences between HY and FO (p < 0.0001) and HO and FO (p<0.0001). Dunn’s multiple comparisons test p values are shown on the graph.

**References**

1. Stroop JR. Studies of interference in serial verbal reactions. J Exp Psychol. 1935;18:643 - 62.

2. Guralnik JM, Simonsick EM, Ferrucci L, Glynn RJ, Berkman LF, Blazer DG, et al. A Short Physical Performance Battery Assessing Lower Extremity Function: Association With Self-Reported Disability and Prediction of Mortality and Nursing Home Admission. Journal of Gerontology. 1994;49(2):M85-M94. <https://doi.org/10.1093/geronj/49.2.M85>

3. Sapey E, Stockley JA, Greenwood H, Ahmad A, Bayley D, Lord JM, et al. Behavioral and structural differences in migrating peripheral neutrophils from patients with chronic obstructive pulmonary disease. Am J Respir Crit Care Med. 2011;183(9):1176-86. DOI: [10.1164/rccm.201008-1285OC](https://doi.org/10.1164/rccm.201008-1285oc)

4. Sapey E, Patel JM, Greenwood H, Walton GM, Grudzinska F, Parekh D, et al. Simvastatin Improves Neutrophil Function and Clinical Outcomes in Pneumonia: a Pilot Randomised Controlled Trial. American Journal of Respiratory and Critical Care Medicine. 2019;200:1282 - 93. DOI: [10.1164/rccm.201812-2328OC](https://doi.org/10.1164/rccm.201812-2328oc)
